# Becoming and staying physically active in adolescents with cerebral palsy: protocol of a qualitative study of facilitators and barriers to physical activity

**DOI:** 10.1186/1471-2431-11-1

**Published:** 2011-01-07

**Authors:** Aniek AOM Claassen, Jan Willem Gorter, Debra Stewart, Olaf Verschuren, Barbara E Galuppi, Lorie J Shimmell

**Affiliations:** 1 CanChild Centre for Childhood Disability Research, McMaster University, Hamilton, Ontario, Canada; 2 School of Rehabilitation Science, McMaster University, Hamilton, Ontario, Canada; 3 Partner of NetChild, Network for Childhood Disability Research in the Netherlands, Rehabilitation Centre De Hoogstraat, Utrecht, the Netherlands; 4 Rehabilitation Centre De Hoogstraat, Centre of Excellence for Rehabilitation Medicine Utrecht, the Netherlands

## Abstract

**Background:**

Adolescents with cerebral palsy (CP) show a reduced physical activity (PA). Currently there are no interventions for adolescents with CP in this critical life phase that optimise and maintain the individuals' physical activity in the long term. To develop such a program it is important to fully understand the factors that influence physical activity behaviours in adolescents with CP. The aim of this study is to explore what makes it easy or hard for adolescents with CP to be and to become physically active.

**Methods/Design:**

A qualitative research method is chosen to allow adolescents to voice their own opinion. Because we will investigate the lived experiences this study has a phenomenological approach. Thirty ambulatory and non-ambulatory adolescents (aged 10-18 years) with CP, classified as level I to IV on the Gross Motor Function Classification System and 30 parents of adolescents with CP will be invited to participate in one of the 6 focus groups or an individual interview. Therapists from all Children's Treatment Centres in Ontario, Canada, will be asked to fill in a survey. Focus groups will be audio- and videotaped and will approximately take 1.5 hours. The focus groups will be conducted by a facilitator and an assistant. In preparation of the focus groups, participants will fill in a demographic form with additional questions on physical activity. The information gathered from these questions and recent research on barriers and facilitators to physical activity will be used as a starting point for the content of the focus groups. Recordings of the focus groups will be transcribed and a content analysis approach will be used to code the transcripts. A preliminary summary of the coded data will be shared with the participants before themes will be refined.

**Discussion:**

This study will help us gain insight and understanding of the participants' experiences and perspectives in PA, which can be of great importance when planning programs aimed at helping them to stay or to become physically active.

## Background

Cerebral Palsy (CP), with a prevalence of 2-3 per 1000 children, is the most common motor disability in paediatric rehabilitation [1]. Because of their motor problems, children and adolescents with CP experience participation restrictions and limitations in physical activities [2,3]. Based on Lelieveld et al. we define physical activities (PA) as all body movements resulting in an increased energy output from the resting position [4]. Children and adolescents with CP show lower levels of PA compared to their healthy peers [5,6]. Lower levels of PA contribute to a reduced physical fitness [5,7], which may increase the risk of developing secondary health problems such as pain and fatigue [8], cardiovascular disease and diabetes mellitus later in life [9]. Moreover, PA is assumed to have a positive relation with health related quality of life and psychosocial functioning [10].

Participation in PA is influenced by age and gross motor function in adolescents with CP. With increasing age, adolescents with CP show a decrease in PA [6,11,12]. Moreover, adolescents with more severe motor problems are participating less in PA than adolescents with less severe motor problems [11,12]. Recent reviews that have evaluated exercise programs in children with CP suggest improvement in muscle strength, coordination and aerobic fitness [13,14]. However, these effects were only partially maintained at follow-up [14] and there is little evidence that these effects carryover into PA and participation [13,14]. Currently there are limited studies to support that current interventions optimise and maintain the individual's PA in the long term [15,16]. We are far from the implementation of promoting PA and changing PA behaviour in practice.

In adolescence, young people develop their adult lifestyle. Health promotion in this developmental phase is critical for all people. Therefore, especially in adolescents with CP, participation in PA should be promoted. Unfortunately, adolescents with CP have to face a discontinuity of care at this stage, as paediatric rehabilitation will end [17]. Current insights suggest that new interventions with a focus on physical fitness and an active lifestyle are important to improve and maintain health in adolescents with CP [18]. The age-specific interventions should primarily address the change in behaviour of adolescents with CP themselves, since from this age adolescents become gradually more autonomous from their parents and develop their personal lifestyle. To develop such an intervention it is important to fully understand the factors that influence PA behaviour in adolescents with CP and factors that make them willing to participate in such programs.

Several studies have been done to investigate barriers to and facilitators of PA in persons with disabilities. In these studies a range of physical, psychological and environmental factors that facilitate or constrain people with disabilities from PA, has been identified [19-22]. Although in one study barriers of and facilitators to PA in adults with CP have been explored [19], no studies have been done to investigate if the same barriers and facilitators exist for adolescents with CP [who are in phases of developing their adult lifestyle]. The present study will build on ongoing research from Olaf Verschuren in the Netherlands, exploring barriers and facilitators of PA in ambulatory children and adolescents with CP, classified at level I and II on the Gross Motor Function Classification System (GMFCS). In the present study we will not only look at the barriers and facilitators, but also at solutions for these barriers. Moreover we will look at ambulatory and non-ambulatory adolescents (10-18 years) classified at level I to IV on the GMFCS in the province of Ontario, Canada.

We aim to develop and evaluate a program to teach people how to promote PA and encourage an active lifestyle in adolescents with CP who are learning to care for themselves; the Stay-FIT study. The present pilot study is a part of the Stay-FIT study. To find out what would be the most optimal Stay-FIT intervention, we need to know more about what issues arise for adolescents with CP, concerning PA. In other words, we need to understand their perspective. Only recently a questionnaire was developed, which focuses on adolescents' opinion about health services, where usually the focus is on adults' or parents' perceptions [23]. Adolescents greatly value their right to make their own decisions and advocate for themselves. Therefore, their views and opinions should be a pivotal starting point in the planning of every program geared towards their health and wellbeing [24]. Nevertheless, most adolescents are still living at home with their family, which makes the role of their parents or caregivers still important. Also the view of therapists in Children's Treatment Centres can be of great value, as they continue to monitor or treat children with CP into their adolescence. The research question of this study is: "What makes it easy or hard for adolescents (age10-18 years), with CP (with a broad range of severity in motor problems) to be and to become physically active?" The main objective is to explore the experience and perspectives of adolescents with CP and their parents. A secondary objective of this study is to survey therapists on facilitators and barriers to physical activity of their clients by using a questionnaire.

## Methods/Design

We explored different methods to find out which method best addressed our research question. Firstly, we considered both quantitative and qualitative approaches. Secondly, different types of qualitative research and qualitative data collection methods were thought over.

### Qualitative versus quantitative research

Quantitative research has the aim to test well-specified hypotheses concerning some predetermined variables [25]. However, before this can be done, these hypotheses need to be generated based on knowledge and theory of the specified topic. We found only two published articles on studies in which barriers to PA in adults with disabilities were investigated using a standardized survey [26,27]. Based on these articles and after contacting the first author of these articles (J.H. Rimmer, personal communication November 2009), we concluded that no such quantitative measures exist for adolescents with CP.

When little is known about a certain topic qualitative research can offer insight into social, emotional, and experiential phenomena in health care and tend not to answer "whether" or "how much" but rather to explore "what", "how", and "why" [25]. Another advantage of qualitative research is that complex phenomena or areas not accessible to quantitative research can be explored [28]. Qualitative research methods allow people to voice their own opinion, rather than conforming to categories and terms imposed on them by others [29].

We concluded that a qualitative approach can be of great use to allow adolescents to voice their opinion, considering we eventually want to develop a PA program for adolescents of different ages, with different preferences and needs. This decision seems appropriate considering the fact that little is known about PA related barriers and facilitators in adolescents with CP and no suitable quantitative measures exist to investigate this concept.

### Types of qualitative research

Qualitative research types are based on different traditions of inquiry and we looked for the best fit between our research question and a specific type. Different research types were explored, such as: a biography, a phenomenology, a grounded theory, ethnography and a case study [30]. A phenomenology can be used for describing the meaning of the lived experiences for several individuals about a concept or phenomenon and seemed most appropriate for our study. Other types that were considered but not chosen were: a grounded theory study with the intent to generate or discover a theory and an ethnography which is a description of and interpretation of a cultural or social group or system.

In conclusion, because the aim of the present study is to investigate the lived experiences of adolescents in PA participation, we will use the phenomenological qualitative approach following the interpretive school, acknowledging presuppositions on PA in adolescents. To our knowledge, no data has been published about adolescents' lived experiences of PA in this manner.

### Data collection methods

The most common types of qualitative data collection methods are field observation (direct observation or indirect observation), interviews (semi-structured, in-depth, individual interviews and focus groups), and document analysis (such as charts, journals and correspondence) [25]. These methods can be used separately or in combination.

To us focus groups make the most sense for data collection for both the exploratory character of the study (What makes it easy or hard for adolescents with CP to be and to become physically active) and the study population (ambulatory and non-ambulatory youth with CP in an age range 10 to 18 years, and parents of adolescents with CP). We will combine the focus groups with additional individual interviews with youth or parents who can contribute in optimizing maximal variation in experiences and perspectives. In summary, we choose a method that would help us to get more a breadth rather than depth in the Stay-FIT pilot study.

Focus groups are semi-structured group meetings, which intend to gather information on a certain topic based on the participants' interaction [31]. Focus groups are unique because they allow the investigator to collect data both from the individual and the individual as part of a larger group [32]. The interaction of group members produces something that is not reducible to individual members or group opinions [32]. They are helpful in understanding how stakeholders regard specific experiences or incidents, fill in gaps in meaning and help understand the 'why' behind attitudes and behaviours [32].

To evaluate therapists' opinions on facilitators and barriers to physical activity of their clients, a questionnaire will be constructed specifically for this purpose. The survey is based on the results of a recent study in the Netherlands on perceived barriers to and facilitators of physical activity in young adults with childhood-onset disabilities [19], expert opinion of the study group members, and feedback from group discussions with physical therapists and occupational therapists from the local Children's Treatment Centre (CTC) "Children's Developmental Rehabilitation Programme (CDRP)" at McMaster Children's Hospital, Hamilton, Ontario, Canada. Therapists of CTC's in the province of Ontario will be invited to complete an online survey.

### Sample selection and recruitment

Adolescents, parents and therapists will be recruited from six Children's Treatment Centres (CTC's) in Ontario who expressed their interest in participating in the Stay-FIT Study and their community network. These centres vary in terms of geography (urban/rural), caseload size, and regional policy planning region.

Adolescents with CP (aged 10-18 years), classified at level I, II, III or IV on the Expanded and Revised-Gross Motor Function Classification System (GMFCS-E&R) [33] will be invited to participate in one of the focus groups or alternatively an individual interview. On behalf of the research team a Site Coordinator in each centre will send out Stay-FIT pilot study informational flyers to adolescents with CP and their parents. Coordinators are instructed to recruit with 'purposeful sampling' strategy in order to include adolescents in different age range and GMFCS level. To maximize the experiences and perspectives coordinators will be encouraged to include both typical and unusual cases with regards to the adolescents' physical activity patterns. These can be, for instance adolescents that are known to be very active or not active at all.

Through the same six CTC's parents of adolescents with CP (aged 10-18 years) will be invited to participate in the study and join one of the focus groups or alternatively an individual interview. To increase variety, we will also include parents of adolescents who are not participating in the Stay-Fit study.

Physical therapists, occupational therapists and recreational therapists from all 20 CTC's in Ontario (see http://www.oacrs.com) with a range of size (large and small) and setting (rural versus urban) will be asked to fill in the survey.

### Sample size

To reach saturation on a certain topic, a minimum of 4 to 5 focus groups is required [31]. With six CTC's participating, we plan to conduct six focus groups in the present study. Saturation will be checked after each meeting. We aim for six to eight participants per focus group as the optimal number. If there are fewer than 5 participants, the dynamics and the interaction of the group will be limited. If there are more than 10 participants there will not be enough time to let every participant express their view [31]. To assure this optimal number of participants we will recruit 12 to 15 participants per focus group. Overall we aim for 30 adolescent participants and 30 parent participants at a minimum. A response rate of 30% of therapists in the CTC's is anticipated based on previous work of our study group.

### Ethics

Ethics approval for this study has been received from the research ethics boards at McMaster University. Two weeks before the focus group, an informed consent or assent form and a demographic form will be sent to the participants.

The information sheets participants will receive contain information about the Stay-FIT pilot study. Information will be given as to why this study is being conducted, what is the participants responsibility in participating in the study, what the possible risks and discomforts are as well as the possible benefits, the rules on privacy, the possibility of not participating or ending participation early and information about costs and payments [Additional File 1]. Because some of the participants are under the age of sixteen, they are not legally authorized to sign their own consent for participating in this study; therefore they will have to sign an assent form. A legally authorized representative will also sign the assent form alongside the signature of the participants that are under sixteen.

### Focus group procedure

The focus groups will be held at the CTC's. A room with adequate size and seating will be arranged for the meetings. Prior to the start of the meetings, the facilitator and the assistant will introduce themselves to the participants and have some informal discussions. Having a little chat is meant to make the participants feel comfortable, but also to get an idea of what the participant is like. The more passive participants will be placed opposite of the facilitator. The more dominant people will be placed next to the facilitator. Before the on-topic discussion will start, an introduction will be given by the facilitator. In this part the purpose of the focus group as part of the Stay-FIT study will be explained.

### Content focus groups Stay-FIT pilot

In preparation of the focus groups, a demographic form with some additional questions on PA will be sent to the participants together with the consent form. Participants will be asked what they consider as PA. They will also be asked what makes it hard and what helps them being physically active, in two open-ended questions. Moreover, a checklist of physical activities will be given. Participant will be asked to check which of activities they did in the last month and what activities they would like to do. The checklist is modified with permission, from the format of the Brunton & Bartlett checklist [11]. The answers to the questions and examples given by the participants will be used as a starting point for the focus group discussions. Examples will be shown on flip-charts and discussed with participants. After the introduction and explaining the definition of PA (Table 1) the main discussion will start. We will address three issues:

**Table 1:** Content of the Stay-FIT pilot focus groups

	Content	
**Definition**	Physical activity: "Any bodily movement resulting in a increased energy output"	
**Discussion**	**Adolescents**	**Parents**
	**Personal factors** • Motivation: - Physical activity is fun - A chance to do things with friends - Energy - Self esteem - Able to do things - Physical activity improves your health/fitness	**Personal factors** • Attitudes towards exercise: - Family preferences - recreation/fitness is a priority for the family - You are physically active yourself - Perception of physical activity - you see physical activity as a positive experience for your child - Fitness benefits - Importance of fitness - Interest - Seeing it as a social opportunity for the child rather than as extra work - Perceived time commitment
	**Environmental factors** • Social support: - Anyone to exercise with (friends/family) - Anyone encouraged you to be active - Anyone told you exercise is important for you • Physical support - Your parents or other people are able to drive you to activities - Transportation is available - Staff able to get the help you need to participate e.g. get changed, use equipment - Are you afraid of hurting yourself • Attitudes - Teasing or bullying - Staring - Embarrassed about how you look or move	**Environmental factors** • Physical support - Transportation and convenience of location and getting to and from activity - Physical support of a family member or program staff - Knowledgeable staff - Perceived safety • Social support - Attitudes of other children/adults - Acceptance - Financial support/subsidized costs • Access to information about available activities/personal guidance for a training plan
		
**Closure**	"Given the discussion on facilitators and barriers, we would like to know what your ideas are for the 'best' Stay-FIT intervention for youth." (Structure, timing, content, setting/location, and people involved, etc.)	

1. What are you doing to be physically active and what helps you to stay physically active?

2. What is keeping you from doing the activities you want to do?

3. What could be a solution for the things that keep you from being physically active?

On the flip-chart the different themes identified from the demographic forms will be showed. There will be one column addressing the things that make it hard to be physically active and one column addressing the things that are helping to be physically active. During the focus group new ideas can be added and solutions can be discussed. In order to build on previous research on barriers to and facilitators of PA, we identified 4 themes for both the focus group with adolescents and the focus group with parents (Table 1). These themes were selected based on previous studies [19-22] and experts experience with adolescents with CP to assure the fit with the adolescent population. At the end of the discussion, these themes will be addressed when they are not mentioned before by the participants.

The focus group discussion will end with an open-ended question related to the aim of the Stay-FIT intervention (Table 1). The participants will explicitly be asked if there are issues or concerns which have not yet been addressed. This is done for the purpose of initial member-checking. Each focus group will take approximately 1.5 hours.

### Focus group Facilitators

A focus group facilitator plays an essential role in conducting the group. In conducting a focus group, a facilitator requires both observational and facilitating skills. These skills help the facilitator to engage all participants in the discussion (both very active participants as the more passive ones), keep the conversation on topic, ensure smooth transition between topics, keep the group enthusiastic, never give his/her own opinion, and all while following the focus group guidelines [31]. In the present study the focus groups will be facilitated by two persons; a facilitator and an assistant. The role of the facilitator is to guide the discussion. The persons that will fulfil the role of facilitator are experienced with facilitating focus groups and are known with the target group. The assistant will make notes, take care of video recordings and add issues on the flipchart. The assistant will also observe the body language of the participants and take note of points that should be further explored.

### Individual interviews

For the individual interviews the same outline as described for the focus groups will be used, allowing the facilitator to get more in-depth on the experiences the participants have with PA. Individual interviews will approximately take 1 hour.

### Data management and analysis

Audiotapes from the video recordings will be transcribed into textual data. The transcripts will be edited for spelling and grammar. Information about participants' identity will be removed. A content analysis approach will be used to analyse the transcripts. This approach involves coding statements based on their key concepts, combining these coded concepts into themes, and refining the identified themes [31].

To ensure accuracy of the data analysis, several procedures will be utilized. These procedures include the purposeful sampling strategy of maximum variation and member checking with the participants on different in-depth levels.

Several transcripts will be read and coded by two researchers independently, after which the coding will be compared among the two researchers. After consensus is reached between the researchers, a final coding scheme will be combined.

The transcripts will be imported into a software program where they can be stored and organized. First, all transcripts will be read by one of the researchers. Second, two other researchers will review the coded transcripts, provide comments and discuss initial interpretations of coded data. Third, the preliminary summary of the coded data will be shared with the participants for the purpose of summary-level member-checking. After this verification, preliminary themes will be refined.

The survey will be described using mean ± SD or frequency distributions. Exploratory analysis will be done for differences among the professionals (physical therapists, recreational therapists and occupational therapists) and CTC's.

## Discussion

The aim of this article was to describe the development and the design of the intended Stay-FIT pilot study. By having focus groups and interviews with adolescents with CP and parents of adolescents with CP and survey therapists, we will gather information about PA from a broad perspective. Changing PA behaviour is complicated. This pilot study will help us gain insight and understanding of the participants' experiences and perspectives in PA. Knowing which barriers need to be overcome and what helps adolescents with CP to be physically active will provide us keystones for what is needed to encourage an active lifestyle. Recent PA interventions including personalized tailored counselling programs seem to have positive effects on physical activity behaviour, through improving attitude and awareness of the importance of a physically active lifestyle [34]. Currently, PA intervention programs for children (7-12) and young people (16-24) with CP that have been developed and are being evaluated in the Netherlands (Learn2Move 7-12 and Learn2Move 16-24) [35,36]. In the Learn2Move interventions individual counselling is included. Results from these evaluations can also be addressed for the development of the Stay-FIT intervention.

In this article a broad description is given about the process of finding the right research method for our research question and the steps taken to set up a qualitative research design. Two recently published reviews on phenomenology and focus group research, underlined the lack of clarification on how this research is actually performed [32,37]. Therefore, this article can be of great value for future research on how to accomplish qualitative research. In paediatric rehabilitation the use of focus groups is relatively new and gaining popularity. Focus groups give adolescents the opportunity to voice their opinions and advocate for themselves. This can be of great importance when planning programs aimed at helping them to stay or to become physically active.

## Competing interests

The authors declare that they have no competing interests.

## Authors' contributions

All authors contributed to the design and the protocol of the study; AAOMC was involved in the design of the study and in drafting the manuscript. JWG is the principal investigator of the Stay-FIT research program. JWG developed the original concept, wrote the grant application, was involved in the design of the study and critically revised the manuscript. DS was involved in the design of the study and critically reviewed the manuscript. OV was involved in the design of the study and critically reviewed the manuscript. BEG is research coordinator of the Stay-FIT pilot study. BEG was involved in the design of the study and critically reviewed the manuscript. LJS was involved in the design of the study and critically reviewed the manuscript. All authors read and approved the final manuscript.

## Pre-publication history

The pre-publication history for this paper can be accessed here:

http://www.biomedcentral.com/1471-2431/11/1/prepub

## Supplementary Material

Additional file 1 **Assent-consent form for adolescents - Stay-FIT pilot study**. This is the informational sheet and the assent-consent form that the adolescents with CP will receive and have to sign in order to participate in the present study. Click here for file

## References

[B1] Surveillance of Cerebral Palsy in Europe Surveillance of cerebral palsy in Europe: a collaboration of cerebral palsy surveys and registers. Surveillance of Cerebral Palsy in Europe (SCPE) Dev Med Child Neurol 2000 42 12 816 824 11132255 10.1017/s0012162200001511

[B2] Imms C Children with cerebral palsy participate: a review of the literature Disabil Rehabil 2008 30 24 1867 1884 10.1080/09638280701673542 19037780

[B3] Donkervoort M Roebroeck M Wiegerink D van der Heijden-Maessen H Stam H Transition Research Group South West Netherlands Determinants of functioning of adolescents and young adults with cerebral palsy Disabil Rehabil 2007 29 6 453 463 10.1080/09638280600836018 17364800

[B4] Lelieveld OT Armbrust W Geertzen JH de Graaf I van Leeuwen MA Sauer PJ van Weert E Bouma J Promoting physical activity in children with juvenile idiopathic arthritis through an internet-based program: results of a pilot randomized controlled trial Arthritis Care Res (Hoboken) 2010 62 5 697 703 10.1002/acr.20085 20191468

[B5] van den Berg-Emons HJ Saris WH de Barbanson DC Westerterp KR Huson A van Baak MA Daily physical activity of schoolchildren with spastic diplegia and of healthy control subjects J Pediatr 1995 127 4 578 584 10.1016/S0022-3476(95)70115-X 7562279

[B6] van Eck M Dallmeijer AJ Beckerman H van den Hoven PA Voorman JM Becher JG Physical activity level and related factors in adolescents with cerebral palsy Pediatr Exerc Sci 2008 20 1 95 106 18364538 10.1123/pes.20.1.95

[B7] Rimmer JH Physical fitness levels of persons with cerebral palsy Dev Med Child Neurol 2001 43 3 208 212 11263693

[B8] Jahnsen R Villien L Stanghelle JK Holm I Fatigue in adults with cerebral palsy in Norway compared with the general population Dev Med Child Neurol 2003 45 5 296 303 10.1111/j.1469-8749.2003.tb00399.x 12729142

[B9] Morris PJ Physical activity recommendations for children and adolescents with chronic disease Curr Sports Med Rep 2008 7 6 353 358 19005359 10.1249/JSR.0b013e31818f0795

[B10] Thorpe D The role of fitness in health and disease: status of adults with cerebral palsy Dev Med Child Neurol 2009 51 Suppl 4 52 58 10.1111/j.1469-8749.2009.03433.x 19740210

[B11] Brunton LK Bartlett DJ Description of exercise participation of adolescents with cerebral palsy across a 4-year period Pediatr Phys Ther 2010 22 2 180 187 10.1097/PEP.0b013e3181db8aaa 20473101

[B12] Orlin MN Palisano RJ Chiarello LA Kang LJ Polansky M Almasri N Maggs J Participation in home, extracurricular, and community activities among children and young people with cerebral palsy Dev Med Child Neurol 2010 52 2 160 166 10.1111/j.1469-8749.2009.03363.x 19549198

[B13] Butler JM Scianni A Ada L Effect of cardiorespiratory training on aerobic fitness and carryover to activity in children with cerebral palsy: a systematic review Int J Rehabil Res 2010 33 2 97 103 10.1097/MRR.0b013e328331c555 19770667

[B14] Verschuren O Ketelaar M Takken T Helders PJ Gorter JW Exercise programs for children with cerebral palsy: a systematic review of the literature Am J Phys Med Rehabil 2008 87 5 404 417 10.1097/PHM.0b013e31815b2675 17993987

[B15] van der Ploeg HP Streppel KR van der Beek AJ van der Woude LH Vollenbroek-Hutten MM van Harten WH van Mechelen W Counselling increases physical activity behaviour nine weeks after rehabilitation Br J Sports Med 2006 40 3 223 229 10.1136/bjsm.2005.021139 16505078 PMC2491980

[B16] van der Ploeg HP Streppel KR van der Beek AJ van der Woude LH Vollenbroek-Hutten MM van Harten WH van Mechelen W Successfully improving physical activity behavior after rehabilitation Am J Health Promot 2007 21 3 153 159 17233232 10.4278/0890-1171-21.3.153

[B17] Roebroeck ME Jahnsen R Carona C Kent RM Chamberlain MA Adult outcomes and lifespan issues for people with childhood-onset physical disability Dev Med Child Neurol 2009 51 8 670 678 10.1111/j.1469-8749.2009.03322.x 19627341

[B18] Fowler EG Kolobe TH Damiano DL Thorpe DE Morgan DW Brunstrom JE Coster WJ Henderson RC Pitetti KH Rimmer JH Rose J Stevenson RD Section on Pediatrics Research Summit Participants, Section on Pediatrics Research Committee Task Force Promotion of physical fitness and prevention of secondary conditions for children with cerebral palsy: section on pediatrics research summit proceedings Phys Ther 2007 87 11 1495 1510 10.2522/ptj.20060116 17895351

[B19] Buffart LM Westendorp T van den Berg-Emons RJ Stam HJ Roebroeck ME Perceived barriers to and facilitators of physical activity in young adults with childhood-onset physical disabilities J Rehabil Med 2009 41 11 881 885 10.2340/16501977-0420 19841838

[B20] Kehn M Kroll T Staying physically active after spinal cord injury: a qualitative exploration of barriers and facilitators to exercise participation BMC Public Health 2009 9 168 10.1186/1471-2458-9-168 19486521 PMC2694784

[B21] Rimmer JH Riley B Wang E Rauworth A Jurkowski J Physical activity participation among persons with disabilities: barriers and facilitators Am J Prev Med 2004 26 5 419 425 10.1016/j.amepre.2004.02.002 15165658

[B22] Vogts N Mackey AH Ameratunga S Stott NS Parent-perceived barriers to participation in children and adolescents with cerebral palsy J Paediatr Child Health 2010 46 11 680 685 10.1111/j.1440-1754.2010.01815.x 20796184

[B23] Siebes RC Wijnroks L Ketelaar M van Schie PE Vermeer A Gorter JW Validation of the Dutch Giving Youth a Voice Questionnaire (GYV-20): a measure of the client-centredness of rehabilitation services from an adolescent perspective Disabil Rehabil 2007 29 5 373 380 10.1080/09638280600835218 17364789

[B24] Gan C Campbell KA Snider A Cohen S Hubbard J Giving Youth a Voice (GYV): a measure of youths' perceptions of the client-centredness of rehabilitation services Can J Occup Ther 2008 75 2 96 104 18510253 10.1177/000841740807500205

[B25] Giacomini MK Cook DJ for the Evidence-Based Medicine Working Group Users' Guides to the Medical Literature: XXIII. Qualitative Research in Health Care A. Are the Results of the Study Valid? JAMA 2000 284 3 357 362 10.1001/jama.284.3.357 10891968

[B26] Rimmer JH Rubin SS Braddock D Barriers to exercise in African American women with physical disabilities Arch Phys Med Rehabil 2000 81 2 182 188 10668772 10.1016/s0003-9993(00)90138-2

[B27] Rimmer JH Wang E Smith D Barriers associated with exercise and community access for individuals with stroke J Rehabil Res Dev 2008 45 2 315 322 10.1682/JRRD.2007.02.0042 18566948

[B28] Pope C van Royen P Baker R Qualitative methods in research on healthcare quality Qual Saf Health Care 2002 11 2 148 152 10.1136/qhc.11.2.148 12448807 PMC1743608

[B29] Sofaer S Qualitative methods: what are they and why use them? Health Serv Res 1999 34 5 Pt 2 1101 1118 10591275 PMC1089055

[B30] Creswell JW Qualitative inquiry and research design. Choosing among five traditions: first ed 1998 United States of America: Sage Publications, Inc

[B31] Crabtree BF Millers WL Doing qualitative research, second edition 1999 second United States of America: Sage Publications, Inc

[B32] Massey OT A proposed model for the analysis and interpretation of focus groups in evaluation research Eval Program Plann 2011 34 1 21 28 10.1016/j.evalprogplan.2010.06.003 20655593

[B33] Palisano RJ Rosenbaum P Bartlett D Livingston MH Content validity of the expanded and revised Gross Motor Function Classification System Dev Med Child Neurol 2008 50 10 744 750 10.1111/j.1469-8749.2008.03089.x 18834387

[B34] van der Ploeg HP Streppel KR van der Beek AJ van der Woude LH van Harten WH van Mechelen W Underlying mechanisms of improving physical activity behavior after rehabilitation Int J Behav Med 2008 15 2 101 108 10.1080/10705500801929684 18569128

[B35] Van Wely LM Becher JG Reinders-Messelink HAP Lindeman E Verschuren OP Verheijden J Dallmeijer AJP LEARN 2 MOVE 7-12 years: a randomized controlled trial on the effects of a physical activity stimulation program in children with cerebral palsy BMC Pediatr 2010 10 1 77 10.1186/1471-2431-10-77 21044314 PMC2989952

[B36] Slaman J Roebroeck ME van Meeteren J van der Slot WM Reinders-Messelink HA Lindeman E Stam HJ van den Berg-Emons RJ LEARN 2 MOVE 16-24: Effectiveness of an intervention to stimulate physical activity and improve physical fitness of adolescents and young adults with spastic cerebral palsy; a randomized controlled trial BMC Pediatr 2010 10 1 79 10.1186/1471-2431-10-79 21054829 PMC2992500

[B37] Norlyk A Harder I What makes a phenomenological study phenomenological? An analysis of peer-reviewed empirical nursing studies Qual Health Res 2010 20 3 420 431 10.1177/1049732309357435 20068190

