# Supplementary material for: Becoming and staying physically active in adolescents with cerebral palsy: protocol of a qualitative study of facilitators and barriers to physical activity
Source: BMC Pediatr. 2011 Jan 7;11:1. doi: 10.1186/1471-2431-11-1 (PMC3025867; doi:10.1186/1471-2431-11-1)
Supplement: Additional file 1 — Assent-consent form for adolescents - Stay-FIT pilot study. This is the informational sheet and the assent-consent form that the adolescents with CP will receive and have to sign in order to participate in the present study. [file 1471-2431-11-1-S1.PDF]

# Physical Activity and Active Lifestyle Program for youth with cerebral palsy:
# A Stay-FIT Pilot Study

## PARTICIPANT INFORMATION SHEET FOR YOUTH

**Local Principal Investigator: Dr. J.W. Gorter, CanChild**

**Co-Investigator(s): Dr. B. Timmons, R. Palisano PhD, V. Wright PhD, Dr. P. Rosenbaum**

**Sponsor:** ***Ontario Federation for cerebral palsy (OFCP)***

---

**WHY IS THIS RESEARCH BEING DONE?**
Youth with cerebral palsy (CP) are often at increased risk for low levels of physical fitness and an inactive lifestyle that can lead to later health problems. After the childhood years young people with CP receive less and less extra care from health care providers to help them keep active. Many experts believe that doctors and therapists need to put more emphasis on including physical activity and fitness into the care of young people with CP. In order to do this, professionals need more information on how best to meet the physical activity and fitness needs of youth and families.

**WHAT IS THE PURPOSE OF THIS STUDY?**
The main purpose of this pilot study is to gather information about how best to meet the physical activity and fitness needs of youth and families.

We plan to develop a program to promote physical activity and encourage an active lifestyle in young people with CP who are learning how to take care of themselves. Once we have enough information, we will want to test out our ideas in a larger intervention study. In order to do this, we first need to understand the things that are working well and also the things that make it difficult for you to be physically active.

In this pilot study, we plan to gather information from 3 different perspectives – from therapists working with youth, from parents of youth and from youth with CP themselves.

**WHAT WILL MY RESPONSIBILITIES BE IF I TAKE PART IN THE STUDY?**
You may be asked to participate in a focus group with other youth with CP or to participate in an individual interview with a research assistant. A focus group is where we get together a group of 5-10 people in person to talk about their perceptions, opinions, beliefs and attitudes towards a particular topic. In this case, it's to talk about your own experiences related to physical activity. It is an interactive group setting where people are invited to share their thoughts and ideas. You can decide how much information you want to share with the group.

You are one of about 30 people who are being asked to participate in a focus group or an individual interview for this pilot study. It will take 1.5 hours and will give you the chance to talk openly about your own experiences. You will also be asked to complete a short survey that will take about 15 minutes to complete. During the focus groups or individual interviews, audio and video recordings will be made and the information gathered will be discussed by members of the study team. You will then be asked to review a summary of the focus group discussion to help the study team be sure that we have understood the ideas correctly, and to give you the chance to add any other thoughts.

**WHAT ARE THE POSSIBLE RISKS AND DISCOMFORTS?**
We do not anticipate any risks or discomforts.

**HOW MANY PEOPLE WILL BE IN THIS STUDY?**
Approximately 30 people will take part in this study.

**WHAT ARE THE POSSIBLE BENEFITS FOR ME AND/OR FOR SOCIETY?**
We cannot promise any personal benefits to you from your participation in this study. However, possible benefits include improvement in health, quality of life and self-management. Your participation may help other people with CP in the future.

**IF I DO NOT WANT TO TAKE PART IN THE STUDY, ARE THERE OTHER CHOICES?**
It is important for you to know that you can choose not to take part in the study. Choosing not to participate in this study will in no way affect your care or treatment.

**WHAT INFORMATION WILL BE KEPT PRIVATE?**
Your data will not be shared with anyone except with your consent or as required by law. All personal information such as your name, address, phone number, will be removed from the data and will be replaced with a number. A list linking the number with your name will be kept in a secure place, separate from your file. The data, with identifying information removed will be securely stored in a locked office in the research office/on a secure server/on an encrypted hard drive, etc. The data for this research study will be retained for 10 years.

For the purposes of ensuring the proper monitoring of the research study, it is possible that a member of the Hamilton Health Sciences/FHS McMaster University Research Ethics Board and a Health Canada representative may consult your research data and medical records. However, no records which identify you by name or initials will be allowed to leave the institution/university/hospital. By signing this consent form, you authorize such access.

If the results of the study are published, your name will not be used and no information that discloses your identity will be released or published without your specific consent to the disclosure.

**CAN PARTICIPATION IN THE STUDY END EARLY?**
If you volunteer to be in this study, you may withdraw at any time. This will in no way affect the quality of care you receive at this institution. You have the option of removing your data from the study. You may also refuse to answer any questions you don't want to answer and still remain in the study. The investigator may withdraw you from this research if circumstances arise which warrant doing so.

**WILL I BE PAID TO PARTICIPATE IN THIS STUDY?**
You will not be paid for participating in this study but a certificate for your volunteer hours will be given.

**WILL THERE BE ANY COSTS?**
Your participation in this research project will not involve any additional costs to you.

**WHAT HAPPENS IF I HAVE A RESEARCH-RELATED INJURY?**
Not applicable.

**IF I HAVE ANY QUESTIONS OR PROBLEMS, WHOM CAN I CALL?**
If you have any questions about the research now or later, you may contact Dr. Gorter at 905-521-2100 x27855.

If you have any questions regarding your rights as a research participant, you may contact the Office of the Chair of the Hamilton Health Sciences/Faculty of Health Sciences Research Ethics Board at 905-521-2100, ext. 42013.

## CONSENT/ASSENT STATEMENT/AUDIO VISUAL RECORDING RELEASE

### SIGNATURE OF RESEARCH PARTICIPANT/LEGALLY-AUTHORIZED REPRESENTATIVE

I have read the preceding information thoroughly. I have had the opportunity to ask questions, and all of my questions have been answered to my satisfaction. I agree to participate in this study. I understand that I will receive a signed copy of this form.

I give permission to McMaster University to make audio and/or video recordings of me. I understand and give permission for the recordings to be used for any of the following: Scientific, or Research purposes of McMaster University (and may be distributed in current or future publications within as well as outside of Canada). I understand that my name will not be identified in the use of the recordings.

I release McMaster University (including their employees, agents, servants and responsible physicians) from all actions, causes of actions, claims and demands resulting from the use of these audio recordings.

**Assent for Youth:**

____________________________________
Name of Participant

____________________________________ ______________
Signature of Participant (or Legally Authorized Representative) Date

**Consent (for Youth under age 16 years):**

____________________________________
Name of Participant

____________________________________
Name of Parent (or Legally Authorized Representative)

____________________________________ ______________
Signature of Parent (or Legally Authorized Representative) Date

**Consent form administered and explained in person by:**

____________________________________
Name and title

____________________________________ ______________
Signature Date
